# *Aldh1a2*^+^ fibroblastic reticular cells regulate lymphocyte recruitment in omental milky spots

**DOI:** 10.1084/jem.20221813

**Published:** 2023-02-28

**Authors:** Tomomi Yoshihara, Yasutaka Okabe

**Affiliations:** 1 Laboratory of Immune Homeostasis, WPI Immunology Frontier Research Center, Osaka University, Osaka, Japan; 2 Center for Infectious Disease Education and Research, Osaka University, Osaka, Japan; 3 Japan Science and Technology Agency, PRESTO, Kawaguchi, Japan

## Abstract

Lymphoid clusters in visceral adipose tissue omentum, known as milky spots, play a central role in the immunological defense in the abdomen. Milky spots exhibit hybrid nature between secondary lymph organs and ectopic lymphoid tissues, yet their development and maturation mechanisms are poorly understood. Here, we identified a subset of fibroblastic reticular cells (FRCs) that are uniquely present in omental milky spots. These FRCs were characterized by the expression of retinoic acid–converting enzyme, *Aldh1a2*, and endothelial cell marker, *Tie2*, in addition to canonical FRC-associated genes. Diphtheria toxin–mediated ablation of *Aldh1a2*^+^ FRCs resulted in the alteration in milky spot structure with a significant reduction in size and cellularity. Mechanistically, *Aldh1a2*^+^ FRCs regulated the display of chemokine CXCL12 on high endothelial venules (HEVs), which recruit blood-borne lymphocytes from circulation. We further found that *Aldh1a2*^+^ FRCs are required for the maintenance of peritoneal lymphocyte composition. These results illustrate the homeostatic roles of FRCs in the formation of non-classical lymphoid tissues.

## Teaser

This study unveils a subset of fibroblastic reticular cells that play an essential role in the formation of milky spots of visceral adipose tissue omentum by regulating lymphocyte recruitment.

## Graphical Abstract

**Figure:**
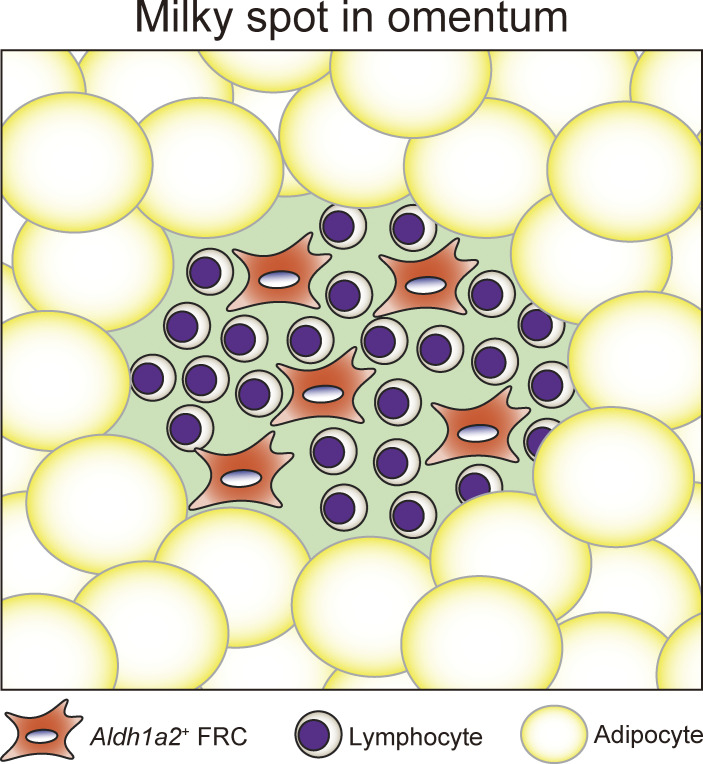

## Introduction

The peritoneal cavity, the largest fluid-filled abdominal cavity in mammals, contains visceral organs such as stomach, spleen, liver, and intestine. Although the peritoneal cavity is normally sterile, infection, which can occur by a pathological or traumatic loss of intestinal wall integrity, cirrhosis, pancreatitis, abdominal surgery, or peritoneal dialysis, can cause life-threatening sepsis since the peritoneal cavity serves as a conduit to other vital organs for pathogen spread (Clements et al., 2021, Vega-Perez et al., 2021). Visceral adipose tissue omentum has important immunological properties against abdominal infection and visceral organ injury (Liu et al., 2021a; Di Nicola, 2019). It is a highly mobile organ that helps to occlude the sites of peritoneal inflammation and injury (Liu et al., 2021b; Zindel et al., 2021). Although it is primarily adipose tissue, the unique feature of omentum is the presence of lymphoid aggregates, termed “milky spots,” which are located beneath the mesothelial surface (Meza-Perez and Randall, 2017). Milky spots compose unique niches to collect antigens, particles, and pathogens in peritoneal cavity and function as places to promote immune responses that in many ways resemble the follicles of secondary lymphoid organs (Koning and Mebius, 2020, Jackson-Jones and Benezech, 2020).

The development of milky spots, similar to secondary lymphoid organs such as lymph nodes and Peyer’s patches, is innately programmed, as the milky spots appear during fetal development in humans, mice, and other mammals (Shimotsuma et al., 1994; Krist et al., 1997; Solvason et al., 1992). Whereas the first phase of lymph node development requires lymphoid tissue–inducer cells that express lymphotoxin-αβ to interact with mesenchymal cells, milky spots do not require these interactions, indicating that the formation of milky spots uses mechanisms distinct from those used by secondary lymphoid organs (Rangel-Moreno et al., 2009). Additionally, postnatal exposure to microbial products greatly increases the size and number of milky spots, which is reminiscent of the formation of tertiary lymphoid organs (Cruz-Migoni and Caamano, 2016; Benezech et al., 2015). These pieces of evidence illustrate the hybrid nature of milky spots between secondary lymphoid organs and ectopic lymphoid tissues; the formation of milky spots is dictated by both hard-wired developmental programs and inflammation-mediated postnatal enhancement. However, the mechanism of milky spot formation is poorly understood.

In addition to triggering peritoneal immunity, milky spots serve as the anatomic site of cell entry into the peritoneal cavity (Meza-Perez and Randall, 2017). Surgical removal of the omentum reduces lymphocyte entry into the peritoneal cavity and abolishes neutrophil influx during the early stage of peritonitis (Berberich et al., 2008; Buscher et al., 2016). Consistently, mice deficient in chemokine CXCL13, an essential chemokine for milky spot formation, and its cognate receptor CXCR5 severely reduce the number of lymphocytes in the peritoneal cavity (Ansel et al., 2002; Rangel-Moreno et al., 2009, Hopken et al., 2004). Milky spots contain specialized blood vessels expressing markers of high endothelial venules (HEVs) that permit entry of blood-borne leukocytes into milky spots (Berberich et al., 2008; Rangel-Moreno et al., 2009). Subsequently, lymphocytes are released to the peritoneal cavity through fenestrations between the mesothelial cells that cover milky spots (Cranshaw and Leak, 1990; Mironov et al., 1979). Thus, the leukocyte transit to the peritoneal cavity, in principle, is regulated by two steps: (1) blood-borne leukocytes entering the milky spots through HEVs and (2) egress to the peritoneal cavity by transmigrating through the mesothelium.

Accumulating evidence indicates that stromal–immune cell interactions are crucial for the development, organization, and function of lymphoid organs (Krishnamurty and Turley, 2020; Krausgruber et al., 2020). Fibroblastic reticular cells (FRCs) are specialized lymphoid organ fibroblasts that form dedicated microenvironments to provide niche factors and regulate immune cell traffic (Lutge et al., 2021; Fletcher et al., 2015). FRCs are characterized by the expression of podoplanin (PDPN) and platelet-derived growth factor receptor-α (PDGFRα, CD140a) and their lack of expression of PECAM-1 and CD45 (Fletcher et al., 2015). They also express molecules common to many myofibroblasts such as desmin, CD105, and secrete chemokine CCL19 (Krishnamurty and Turley, 2020, Lutge et al., 2021). In classical secondary lymphoid organs, subsets of FRCs including T cell zone FRCs, B cell zone FRCs, pericytic FRCs, marginal reticular cells, and follicular dendritic cells have been identified based on their localization and phenotypic markers, each of which performs dedicated functions to underpin lymphoid organ microarchitecture (Li et al., 2021). On the contrary, milky spots of omentum display a reduced structural complexity lacking neither clear segregation of B and T cell zones nor a well-defined follicular dendritic cell network (Rangel-Moreno et al., 2009; Perez-Shibayama et al., 2019). Although it has emerged that FRCs in milky spots are crucial for triggering abdominal immune responses (Perez-Shibayama et al., 2018), understanding of phenotypic and functional diversity of FRCs in the milky spots is under-explored. Here, we identified a novel subset of milky spot FRCs, characterized by the expression of retinoic acid–converting enzyme, *Aldh1a2*, as well as the endothelial cell marker, *Tie2*. Genetic depletion of *Aldh1a2*^+^ FRCs led to a substantial reduction in the number of T cells and conventional B2 B cells in milky spots due to the impaired recruitment of these lymphocytes from circulation. Mechanistically, we found that *Aldh1a2*^+^ FRCs are required for the induction of homeostatic chemokine CXCL12 displayed on HEVs in a manner dependent on retinoic acid. Lastly, a progressive decline in the number of lymphocytes in the peritoneal cavity was observed by *Aldh1a2*^+^ FRC ablation. Altogether, these results demonstrate a subset of FRCs that are an integral component of milky spot formation through the regulation of lymphocyte recruitment.

## Results

### Identification of ALDH1A2-expressing stromal cells in milky spots

**Figure 1.**
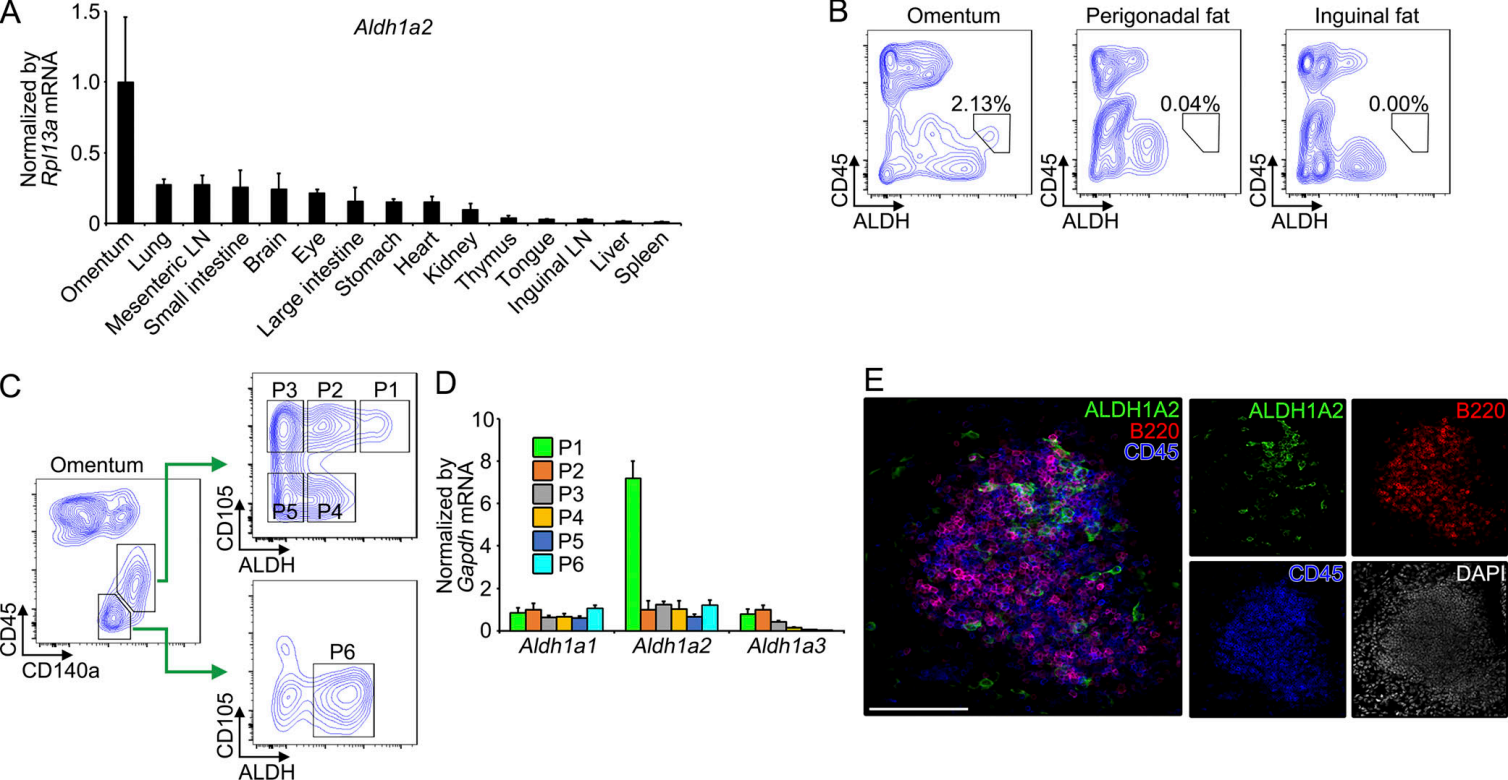
**Identification of ALDH1A2-expressing stromal cells in milky spots. (A)** Indicated tissues were determined for *Aldh1a2* mRNA by quantitative PCR and expressed as relative values normalized by *Rpl13a* mRNA. **(B)** ALDH activity of total cells isolated from omentum (50,283 cells), perigonadal fat (41,885 cells), and inguinal fat (32,335 cells) was determined by flow cytometry. **(C)** Flow cytometry analysis of omental cells (51,021 cells) and gating strategy of cell populations (P1–P6) were depicted. **(D)** The expression of the indicated genes encoding retinaldehyde dehydrogenases in omental cell populations (P1–P6) were analyzed by quantitative PCR and normalized by *Gapdh* mRNA. Expression levels were shown as relative values compared to that of P2. **(E)** Milky spot of the whole-mount omuntum from WT mouse was analyzed for ALDH1A2, B220, CD45, and DAPI. Data represent means ± SEM of three mice per group (A and D), or representative flow cytometry plot or images from at least two independent experiments with similar results (B, C, and E). Scale bar: 100 μm (E).

**Figure S1.**
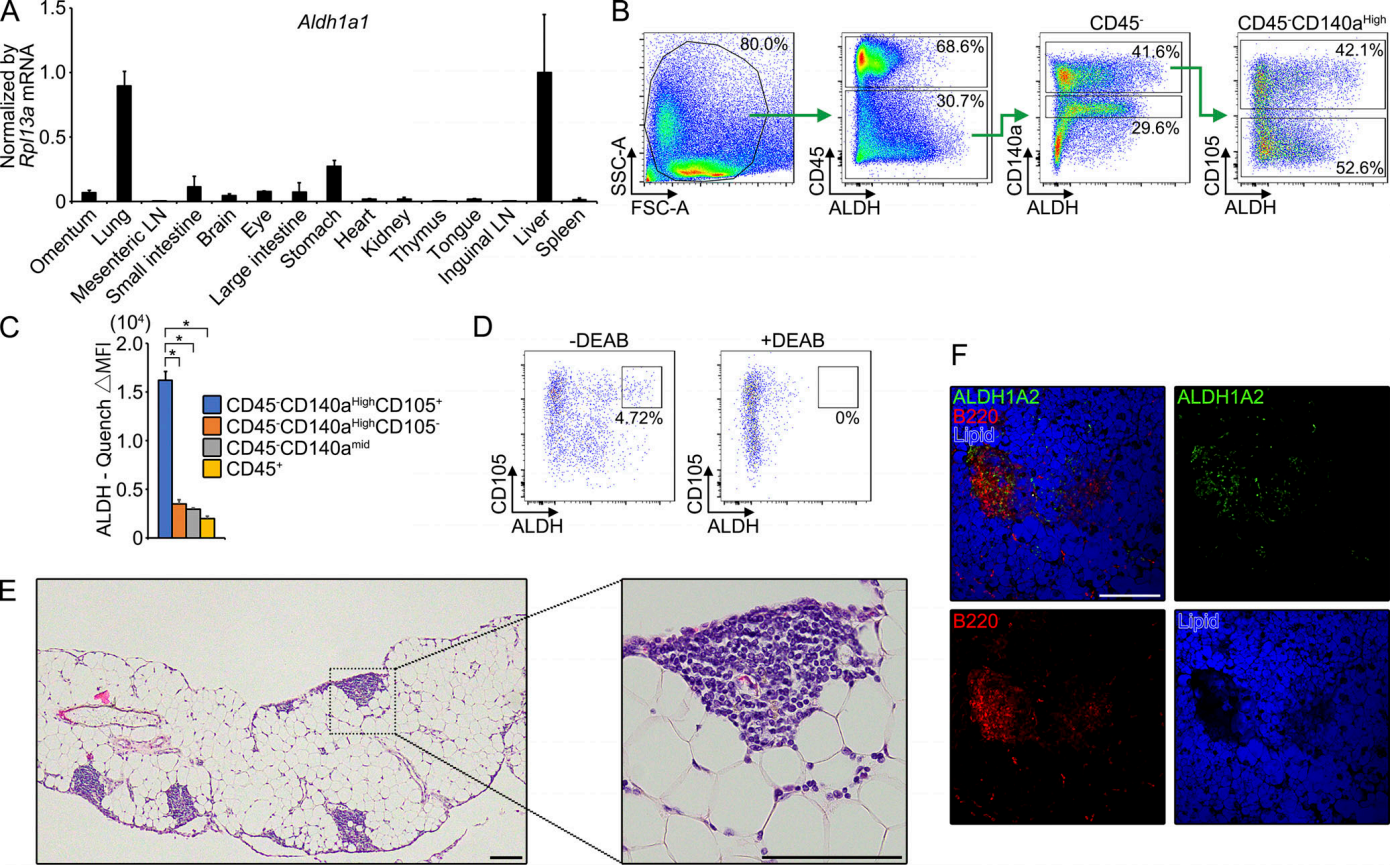
**Characterization of cells exhibiting retinoic acid–producing capacity in omentum. (A)** Indicated tissues were determined for *Aldh1a1* mRNA by quantitative PCR and were expressed as relative values normalized by *Rpl13*a mRNA. **(B)** Gating strategy used for flow cytometry of omental single-cell suspension (157,388 cells analyzed) to identify cells exhibiting high ALDH activity. **(C)** Aldefluor activity (aldefluor mean fluorescence intensity [MFI] minus quench MFI) across omental stromal populations in B is shown (*n* = 4). **(D)** CD45^−^CD140a^High^ omental cells were treated with ALDH in the presence (right) or absence (left) of inhibitor of the retinoic acid production pathway, diethylaminobenzaldehyde (DEAB). **(E)** Paraffin sections of omentum from WT mouse were stained with hematoxylin and eosin. **(F)** Milky spots of the whole-mount omuntum from WT mouse were shown as indicated color-coded lettering. Data represent means ± SEM of three (A) or four (C) mice per group, or representative flow cytometry plot or images from at least two independent experiments (B, E, and F). P value <0.05 was considered significant (*, P < 0.0001). One-way ANOVA was used. Scale bar: 100 μm (E), 200 μm (F).

Multiple studies have shown that retinoic acid, a lipophilic molecule derived from vitamin A, is abundantly present in omentum, and we previously showed that retinoic acid regulates functional polarization of macrophages through the induction of transcription factor GATA6 (Maruya et al., 2011; Okabe and Medzhitov, 2014; Jayakumar et al., 2022). Retinoic acid controls a broad array of immune functions including lymphocyte/myeloid cell differentiation, class switching of B cells, lymphocyte homing, and lymphoid organization (Erkelens and Mebius, 2017), suggesting the role(s) of retinoic acid not only in macrophage regulation but also in other immunological functions in omentum. Omentum was characterized by the high expression of *Aldh1a2* gene, which encodes a rate-limiting enzyme regulating retinoic acid synthesis, whereas *Aldh1a1*, another important enzyme for retinoic acid synthesis, showed a distinct expression pattern (Fig. 1 A and Fig. S1 A). Although previous studies reported that mesothelial cells and fibroblasts in omentum expressed *Aldh1a2* gene (Buechler et al., 2019; Jackson-Jones et al., 2020), the cellular characterization and contribution of each cell type in omental biology remains to be determined. To characterize *Aldh1a2*-expressing cells in omentum, we utilized fluorescent substrate for aldehyde dehydrogenase (ALDH) enzymes, which assesses retinol metabolism capacity (Buechler et al., 2019). Whereas ALDH activity was detected in non-hematopoietic cells (CD45^−^) of all adipose tissues (omentum, perigonadal, and inguinal fat), we found a fraction of CD45^−^ cells in omentum exhibited the highest ALDH activity (Fig. 1 B). This fraction was further identified as CD45^−^CD140a^+^CD105^+^ALDH^high^ cells (Fig. 1 C and Fig. S1, B–D). In this population (gated as P1 in Fig. 1 C), the expression of *Aldh1a2* mRNA, but not two other retinaldehyde dehydrogenases (*Aldh1a1* and *Aldh1a3*), was enriched (Fig. 1 D), suggesting that selective expression of *Aldh1a2* endows the strong ALDH activity. We next determined the localization of ALDH1A2-expressing cells in omentum. Omentum was predominantly occupied by adipose tissue in which scattered distribution of milky spots was observed (Fig. S1 E). Immunofluorescence staining of whole-mount omentum revealed that the majority of cells expressing ALDH1A2 were found to localize in milky spots (Fig. 1 E and Fig. S1 F). These results indicate that a fraction of stromal cells in milky spots exhibit high retinoic acid–metabolizing capacity via the expression *Aldh1a2* gene.

### Cellular characterization of ALDH1A2-expressing cells

**Figure 2.**
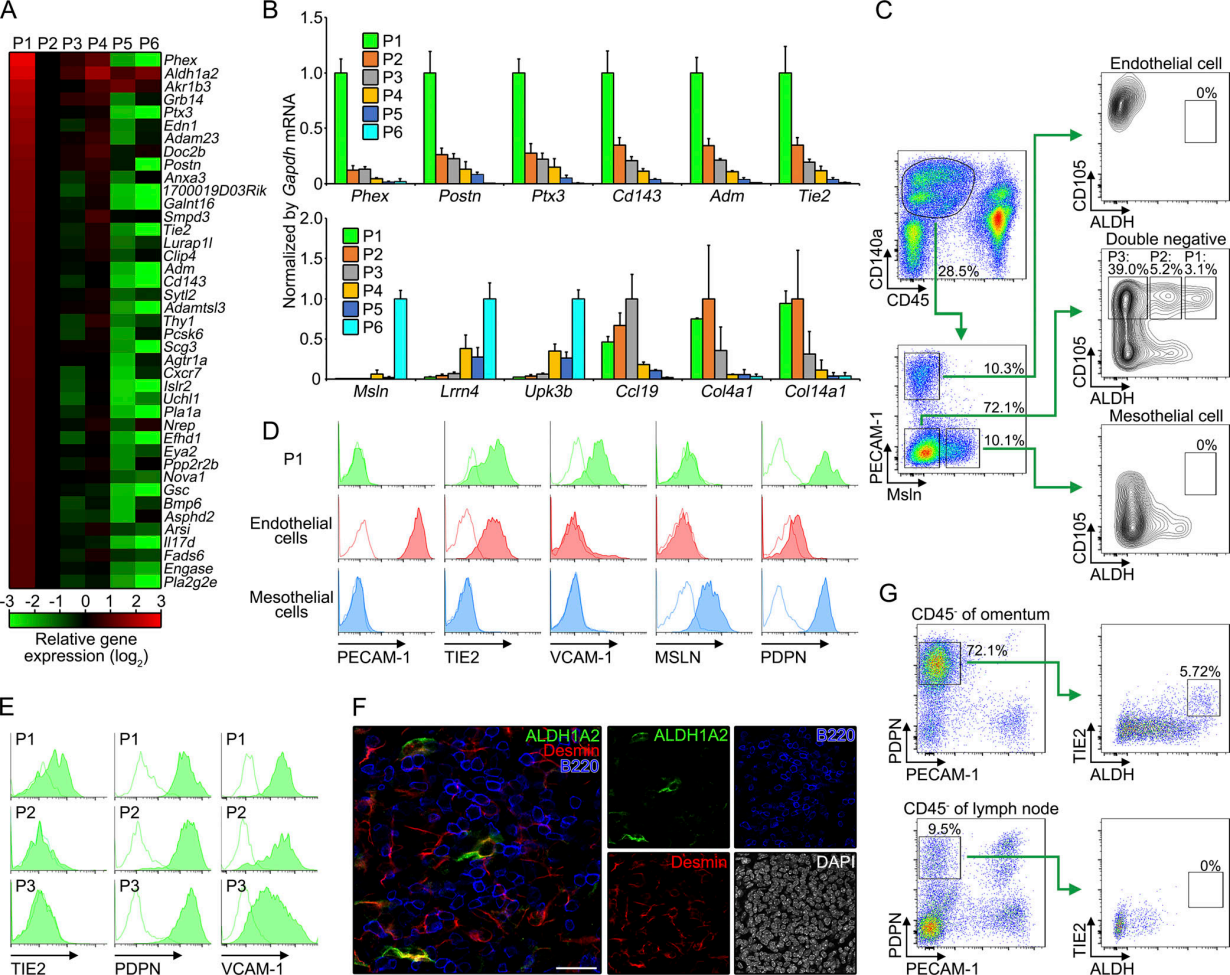
**Cellular characterization of *Aldh1a2***^**+**^ **stromal cells. (A)** Heat map of mRNA, as measured by RNA-seq, expressed at least two times over in P1 relative to their expression in the remaining five populations. Expression levels were shown as relative values normalized by that of P2. **(B)** The mRNA expression of the genes highly expressed in P1 (upper), and mesothelial and FRC markers (lower) in P1–P6 was determined by quantitative PCR and is expressed as a relative value to *Gapdh* mRNA. **(C)** Flow cytometric gating strategy for P1, P2, P3, mesothelial cells, and endothelial cells of WT omental cells (171,123 cells). **(D and E)** Expression of indicated proteins in P1, mesothelial cells, and endothelial cells (D) or omental FRCs (E) was determined by flow cytometry. Dotted lines indicate isotype controls. **(F)** Milky spot of the omuntum from WT mouse was analyzed for ALDH1A2, B220, Desmin, and DAPI. **(G)** Omentum (374,931 cells) and inguinal lymph node (3,877,416 cells) were analyzed for CD45^−^PDPN^+^PECAM-1^−^ FRCs, and determined TIE2 expression and ALDH by flow cytometry. Data represent means ± SEM of three mice per group (B) or representative flow cytometry plot or images from at least two independent experiments (C–G). Scale bar: 20 μm (F).

**Figure S2.**
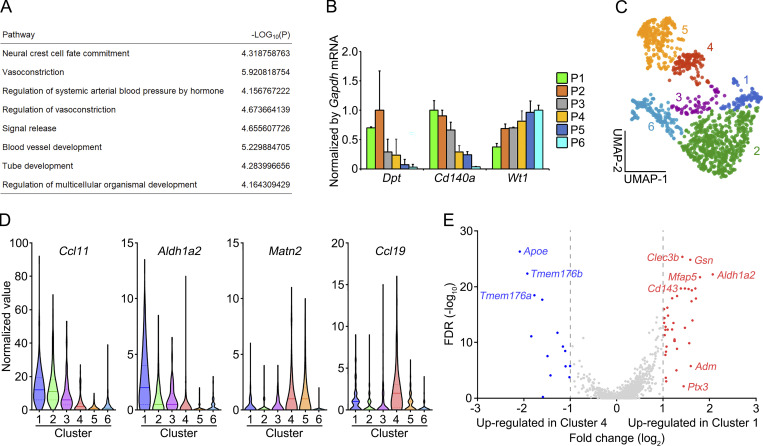
**Gene expression analysis of *Aldh1a2*^+^ stromal cells. (A)** Gene ontology enrichment analysis of a set of genes that were selectively expressed in P1 compared with the other fractions (P2–P6). **(B)** The mRNA expression of indicated genes was determined by quantitative PCR and is expressed as a relative value to *Gapdh* mRNA (*n* = 3). **(C)** Uniform Manifold Approximation and Projection clustering of scRNA-seq of CD140a^+^ omental stromal cells (CD45^−^CD41^−^TER119^−^PCAM1^−^PDPN^+/−^) obtained from Jackson-Jones et al. (2020). **(D)** Violin plots of indicated genes by clusters. Median and first and third quartile are shown by lines and dot lines, respectively. **(E)** Volcano plots of differentially expressed genes between Cluster 1 and Cluster 4. The x axis refers to the log_2_ fold-change of the gene in Cluster 1 compared with Cluster 4, while the y axis shows the −log_10_ Benjamini–Hochberg adjusted P value. Genes with log fold-change >1 or > −1 are colored red or blue, respectively.

To characterize the cellular phenotype of *Aldh1a2*-expressing cells, we next performed RNA sequencing (RNA-seq) analysis on sorted omental stromal populations (P1–P6 in Fig. 1 C). We identified 41 genes that had the expression of at least twofold higher in P1 compared to all five other populations (Fig. 2 A), and the selective expression of *Phex*, *Postn*, *Ptx3*, *Cd143*, *Adm*, and *Tie2* in P1 was confirmed by quantitative PCR (Fig. 2 B). Gene ontology analysis revealed that highly expressed genes in P1 are associated with endothelial phenotypes including “vasocontraction” and “blood vessel development” (Fig. S2 A), whereas P1 was clearly distinguishable from PECAM-1^+^ endothelial cells that exhibited low ALDH activity (Fig. 2 C). In contrast, P1 constitutively expressed other well-defined endothelial cell markers, TIE2 and VCAM-1 (Fig. 2 D; Goncharov et al., 2020), suggesting a phenotypic link between P1 and endothelial cells. We also found P1-expressed PDPN, which is known to be expressed in lymphatic endothelial cells, mesothelial cells, and FRCs (Fig. 2 D). Meanwhile, the expression of mesothelial cell markers *MSLN*, *Lrrn4*, and *Upk3b* was not detected in P1 at protein and mRNA levels (Fig. 2, B and D), indicating that P1 does not represent the cells that are generally considered mesothelial cells.

In secondary lymphoid organs, PDPN^+^PECAM-1^−^CD140a^+^VCAM-1^+^CD105^+^ cells, which correspond to P1, P2, and P3 (Fig. 2, C and E), are generally referred to FRCs (Perez-Shibayama et al., 2018). Indeed, they were enriched in the expression of FRC-associated genes including *Ccl19*, *Col4a1*, *Col14a1*, *Dpt*, and *Cd140a* (Fig. 2 B and Fig. S2 B). Additionally, ALDH1A2^+^ cells were found in Desmin^+^ filamentous network of FRCs in milky spots (Fig. 2 F; Zeng et al., 2011). These results indicate that omental *Aldh1a2*^+^ cells are a subset of FRCs. Although the expression of FRC-associated genes was also detected in P2 and P3 at mRNA and protein levels, TIE2 was exclusively expressed in P1 (Fig. 2 E). Notably, the cells displaying both ALDH activity and TIE2 were absent among FRCs (CD45^−^PDPN^+^PECAM-1^−^) in lymph nodes, suggesting that *Aldh1a2*^+^ cells represent a subset of FRCs distinct from the cells that are present in secondary lymphoid organs. To further delineate the cellular phenotype of *Aldh1a2*^+^ FRCs, we analyzed single-cell RNA-seq (scRNA-seq) data on *Cd140a*^+^ cells of omental CD45^−^CD41^−^TER119^−^PECAM1^−^PDPN^+/−^ stromal cells (Fig. S2 C; obtained from Jackson-Jones et al., 2020). We found *Aldh1a2* expression was enriched in a fraction of *Ccl11*^+^*CD140a*^+^ fibroblasts (Cluster 1 in Fig. S2, C and D), which was distinct from previously described omental FRCs that were characterized by the expression of *Ccl19*, *Matn2*, and *CD140a* (Cluster 4 in Fig. S2, C and D). Consistent with RNA expression analysis (Fig. 2 B), *Aldh1a2*^+^*Ccl11*^+^*CD140a*^+^ cluster was enriched with the expression of *Ptx3*, *Cd143*, and *Adm*, supporting that this cluster corresponded to *Aldh1a2*^+^ FRCs (Fig. S2 E). Moreover, we identified genes that were highly expressed in *Aldh1a2*^+^*Ccl11*^+^*CD140a*^+^ cluster including *Clec3b*, *Gsn*, and *Mfap5* (Fig. S1 E). These results suggest that at least two distinct FRC subsets, *Aldh1a2*^+^ FRCs and *Ccl19*^+^ FRCs, are present in omentum.

### In vivo ablation of *Aldh1a2*^+^ FRCs

**Figure S3.**
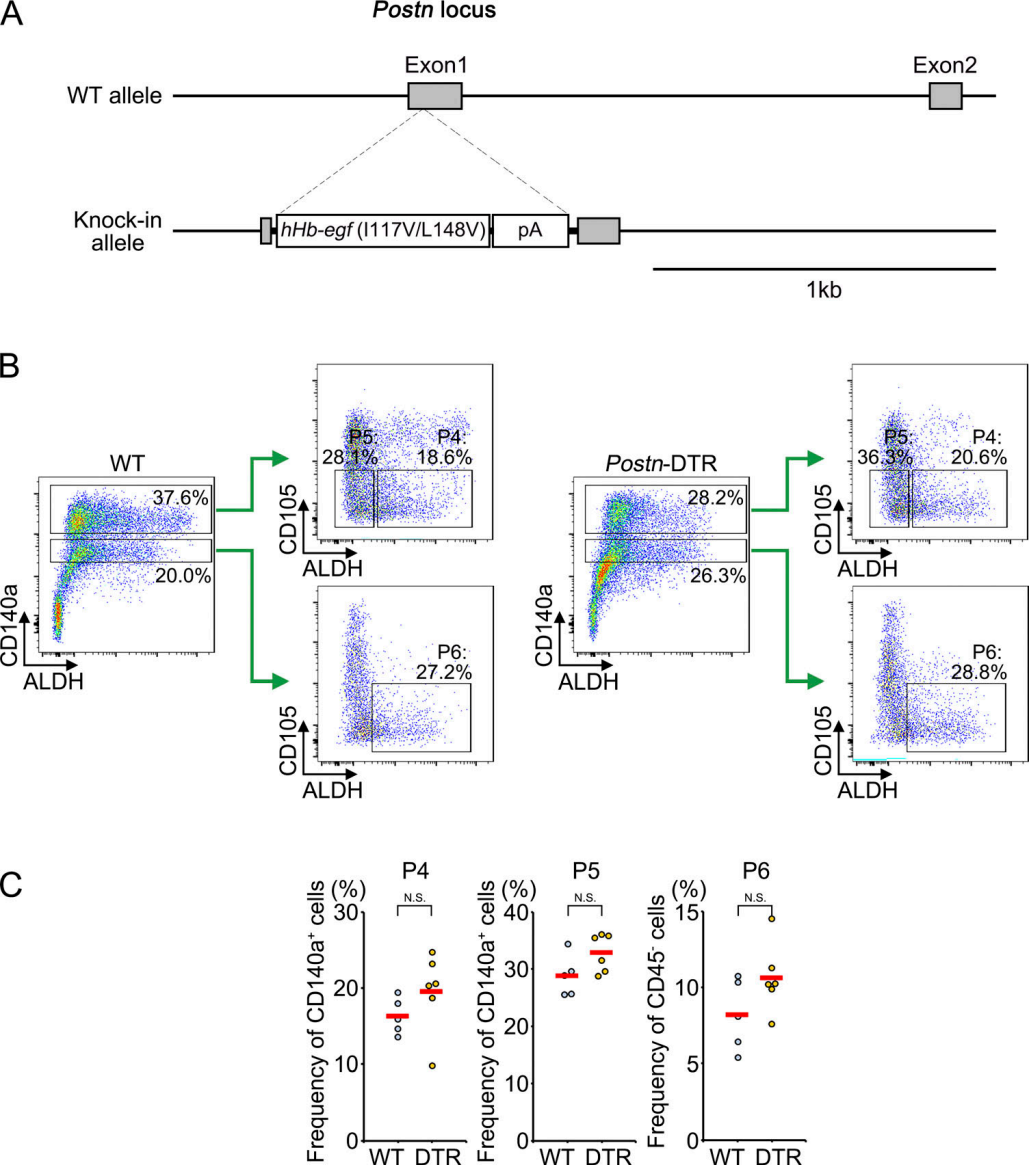
**Generation of *Postn*-DTR mice. (A)** Targeting strategy for generating *Postn*-DTR mouse. Microinjection-based generation of *Postn*-DTR allele by targeting insertion of a DTR cassette (h*Hb-egf*, I117V/L148V) by the CRISPR/Cas9 system. **(B)** Omenta of WT and *Postn*-DTR mice were analyzed for P4, P5, and P6 at 24 h after DT injection. **(C)** The frequencies of P4, P5, and P6 in B were shown. Each point represents one mouse, and the mean values were shown by red horizontal lines (*n* = 5–6). P value <0.05 was considered significant. N.S., not significant. Student’s *t* test was used.

**Figure 3.**
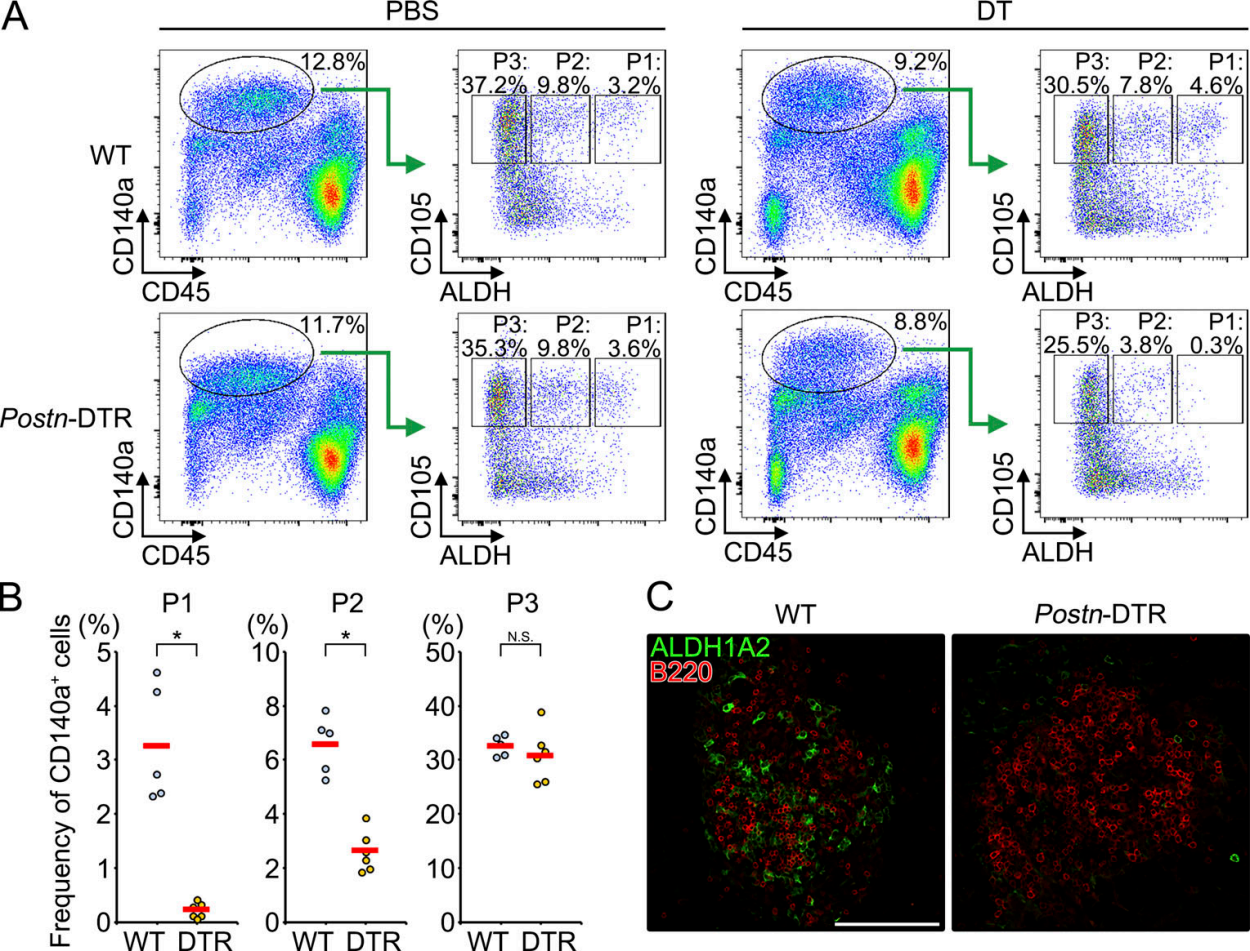
**Genetic depletion of *Aldh1a2***^**+**^ **FRCs. (A)** WT and *Postn*-DTR mice were i.p. injected with PBS or 0.1 μg DT. Omenta were analyzed by flow cytometry 24 h after injection. **(B)** The frequencies of P1, P2, and P3 in A were shown. Each point represents one mouse and the mean values were shown by red horizontal lines (*n* = 5–6). **(C)** Whole-mount omenta from indicated mice (WT or *Postn*-DTR) were stained as indicated color-coded lettering. Data are representative flow cytometry plot or images from at least two independent experiments (A and C). P value <0.05 was considered significant (*, P < 0.0001). N.S., not significant. Student’s *t* test was used. Scale bar: 100 μm (C).

Among the genes that are selectively expressed in P1 (Fig. 2 B), Periostin (*Postn*) is known to be expressed in restricted cell lineages including myofibroblasts, osteoblasts, and colonic epithelial cells (Kanisicak et al., 2016). To determine in vivo function(s) of *Aldh1a2*^+^ FRCs, we utilized *Postn* promoter to selectively target *Aldh1a2*^+^ FRCs in the omentum and generated mice in which a diphtheria toxin receptor (DTR) was genetically targeted into the first coding exon of *Postn* gene (Fig. S3 A; Furukawa et al., 2006). Heterozygous mice were healthy and born at the expected Mendelian ratio, and we hereafter used *Postn*^Dtr/+^ heterozygotes for experiments (referred to as *Postn*-DTR mice). A single injection of low-dose (100 ng/mouse) diphtheria toxin (DT) through the i.p. route efficiently ablated P1 (3.1 ± 0.8% for WT vs. 0.4 ± 0.2% for *Postn*-DTR among CD140a^+^ population; Fig. 3, A and B). While DT administration had only marginal effect on the frequency of P3–P6, it ablated P2 to some degree (6.7 ± 0.8% for WT vs. 3.5 ± 0.7% for *Postn*-DTR among CD140a^+^ population; Fig. 3 B and Fig. S3, B and C), suggesting that P2 could be precursors of P1 or vice versa. The depletion of ALDH1A2^+^ FRCs in the milky spots was confirmed by immunofluorescence staining (Fig. 3 C). These results indicate that DT injection in *Postn*-DTR mice selectively ablated *Aldh1a2*^+^ FRCs.

### *Aldh1a2*^+^ FRCs induce the recruitment of circulating lymphocytes

**Figure 4.**
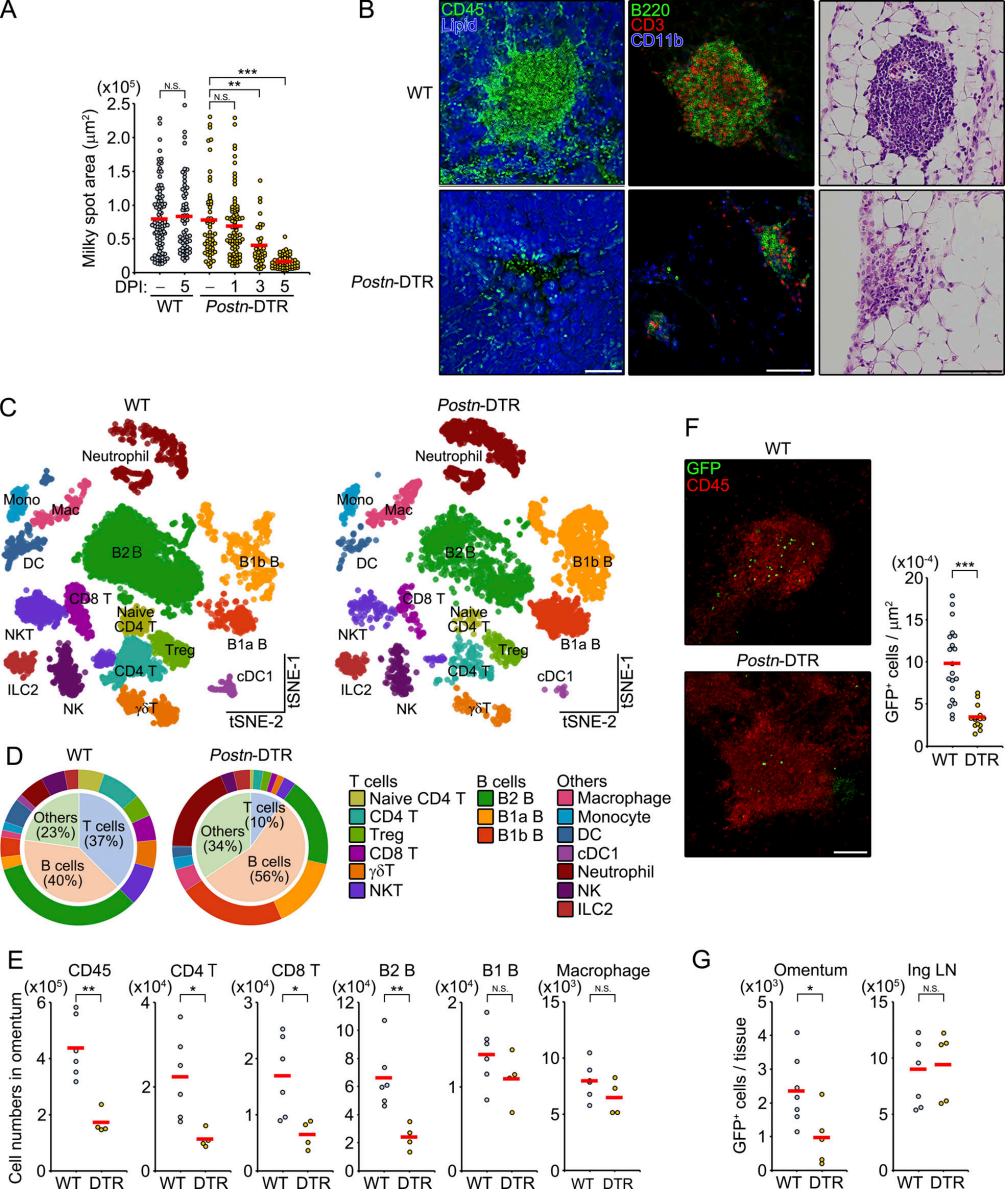
***Aldh1a2***^**+**^ **FRCs regulate the cellularity of milky spots. (A)** Whole-mount omenta from WT and *Postn*-DTR mice at 1, 3, and 5 d after PBS or DT injection (DPI) were visualized by cumulative vertical images and the area of milky spots was determined. Each plot represents one CD45^+^ lymphoid aggregate. Red horizontal lines indicate the mean. **(B)** WT and *Postn*-DTR mice were i.p. injected with DT, and representative images of milky spots at 5 d after DT injection were shown as indicated color-coded lettering (left and middle) or with hematoxylin and eosin (right). **(C)** t-SNE visualization of color-coded scRNA-seq omental CD45^+^ hematopoietic populations of WT and *Postn*-DTR mice at 5 d after DT injection. **(D)** Pie charts demonstrate proportions of the identified clusters across samples. **(E)** Numbers of CD45 cells, CD4 T, CD8 T, B2 B, B1 B, and macrophages in the omentum from WT and *Postn*-DTR mice at 5 d after DT injection (*n* = 4–6). **(F)** GFP^+^ splenocytes were i.v. transferred into WT or *Postn*-DTR mice at 24 h after DT injection, and donor GFP^+^ cells in the milky spots were determined 6 h after transfer. Representative images of GFP^+^ cells in the milky spots were shown (left) and the numbers of GFP^+^ cells in the milky spots were determined (right). Each plot represents a single milky spot, and red horizontal lines indicate the mean. **(G)** The numbers of GFP^+^ cells in omentum and inguinal lymph node (Ing LN) in F were determined by flow cytometry. Pooled data from at least two independent experiments (A, E, and G) or data representative of at least two independent experiments with similar results (B and F) are shown. P value <0.05 was considered significant (*, P < 0.05; **, P < 0.01; ***, P< 0.0001). N.S., not significant. One-way ANOVA was used for A, and Student’s *t* test was used for E–G. Scale bar: 100 μm (B and F). DC, dendritic cell; cDC1, conventional dendritic cell 1; NK, natural killer cell; ILC2, innate lymphoid cells; Treg, regulatory T cell.

**Figure S4.**
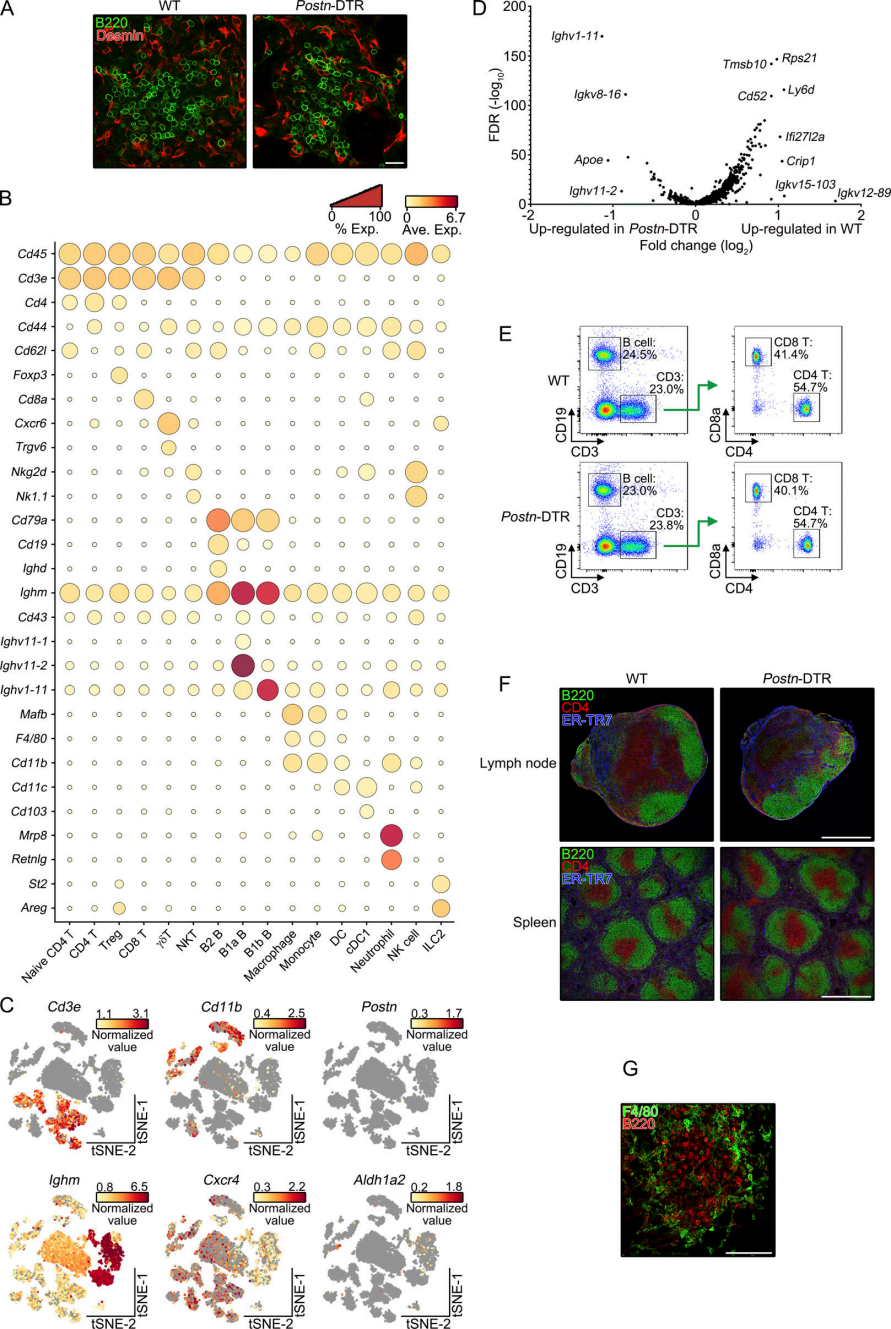
**Characterization of leukocyte populations in *Postn*-DTR mice. (A)** Immunofluorescence staining of whole-mount omentum of WT and *Postn*-DTR mice at 5 d after DT injection demonstrating the expression of Desmin and B220. **(B)** Dot plots demonstrating scaled gene expression and percentage of cells expressing these genes for cluster phenotyping markers for CD45^+^ hematopoietic populations. **(C)** The expression of indicated genes was projected onto t-SNE plots. Color scaled for each gene with highest normalized expression level noted. **(D)** Volcano plot depicting relative gene expression change of CD4 T, CD8 T, and B2 B cells between WT and *Postn*-DTR mice. **(E)** B, CD4 T, and CD8 T cells in peripheral blood from WT and *Postn*-DTR mice were analyzed at 5 d after DT injection. **(F)** Inguinal lymph node and spleen of WT or *Postn*-DTR mice were analyzed for B220, CD4, and ER-TR7 at 5 d after DT injection. **(G)** Milky spot of the whole-mount omuntum from WT mouse was analyzed for F4/80 and B220. Representative flow cytometry plot (E) or images (A, F, and G) from at least two independent experiments are shown. Scale bar: 20 μm (A), 500 μm (F), 100 μm (G).

Given that FRCs are an integral component of the organization and function of the lymphoid organs (Acton et al., 2021; Cremasco et al., 2014; Kapoor et al., 2021), we next explored whether the ablation of *Aldh1a2*^+^ FRCs affected the structure of milky spots. To this end, we performed immunostaining of whole-mount omenta with anti-CD45 antibody and the area of CD45^+^ cell aggregates was determined by Z-stack imaging. Although no obvious change in the size of milky spots was observed at 1 d after DT injection, *Postn*-DTR mice severely reduced the size by 5 d after DT administration (Fig. 4, A and B, left). Moreover, the milky spots of *Postn*-DTR mice showed low follicle density (Fig. 4 B, right) and possessed fewer numbers of B220^+^ or CD3^+^ lymphocytes than that of WT mice (Fig. 4 B, middle). In contrast, Desmin^+^ network was still observed in *Postn*-DTR mice (Fig. S4 A). To further assess the alteration to omental leukocyte populations, we performed an scRNA-seq of cells isolated from omenta of WT and *Postn*-DTR mice at 5 d after DT injection (three pooled mice per group). After quality control and adjusting for technical noise, CD45^+^ hematopoietic cells (7,997 cells for WT mice and 5,233 cells for *Postn*-DTR mice) were determined by unsupervised classification visualized by using *t*-distributed stochastic neighbor embedding (t-SNE), and we identified 16 cell types based on the expression of canonical cell marker genes (Fig. 4 C and Fig. S4, B and C). Omental leukocytes were predominantly composed of T and B lymphocytes that were designated with six T cell subsets including naive CD4 T cells (*Cd3e*^+^*Cd4*^+^*Cd62l*^+^*Cd44*^−^), CD4 T cells (*Cd3e*^+^*Cd4*^+^*Cd62l*^−^*Cd44*^+^), regulatory T cells (*Cd3e*^+^*Cd4*^+^*Foxp3*^+^), CD8 T cells (*Cd3e*^+^*Cd8a*^+^), γδT cells (*Cd3e*^+^*Cxcr6*^+^*Trgv6*^+^), and natural killer T cells (*Cd3e*^+^*Nkg2d*^+^ *Nk1.1*^+^), and three B cell subsets that were B2 B cells (*Cd19*^+^*Cd79a*^+^*Ighd*^+^), B1a B cells (*Ighm*^+^*Cd43*^+^*Ighv11-1*^+^*Ighv11-2*^+^), and B1b B cells (*Ighm*^+^*Cd43*^+^*Ighv1-11*^+^; Barington et al., 2022; Prohaska et al., 2018; Fig. 4 C). Among them, the proportion of all T cell subsets was markedly reduced in *Postn*-DTR mice (Fig. 4 D). The proportion of conventional B2 B cells was also reduced in *Postn*-DTR mice whereas that of B1a and B1b B cells showed the opposite trend (Fig. 4 D). Consistently, *Postn*-DTR mice showed a substantial reduction in the cell numbers of CD4 T, CD8 T, and B2 B cells as well as total CD45^+^ leukocytes, although that of B1 B cells and macrophages was not altered (Fig. 4 E). These results suggest that ablation of *Aldh1a2*^+^ FRC led to the selective loss of lymphocyte subsets in omentum. We also found that genes such as *Rps21*, *Tmsb10*, *Ly6d*, and *Cd52* were highly expressed in T cells and B2 B cells of WT mice compared with that of *Postn*-DTR mice, as illustrated in the volcano plot (Fig. S4 D). Interestingly, *Cd52* has been shown to be induced by retinoic acid in lymphocytes (Li et al., 2003), suggesting the involvement of retinoic acid in the functionality and activation of these lymphocytes. The reduction of these lymphocyte numbers in omenta of *Postn*-DTR mice was unlikely due to off-target depletion of lymphocytes since *Postn* was barely detected in omental leukocytes under homeostatic settings (Fig. S4 C), DT did not affect the frequency of circulating lymphocytes in peripheral blood (Fig. S4 E), and lymphoid structures and ER-TR7^+^ fibroblastic network of lymph nodes and spleen were intact in *Postn*-DTR mice (Fig. S4 F).

T cells and B2 B cells constitutively recirculate between the omentum and other lymphoid tissues, whereas macrophages and B1 B cells are permanent residents in omentum and peritoneal cavity and recirculate with relatively slow rate, respectively (Ansel et al., 2002; Berberich et al., 2008). This suggested that the selective reduction of T cell and B2 B cell numbers in *Postn*-DTR omenta was possibly due to the impaired recruitment of circulating lymphocytes to milky spots. To test this possibility, GFP-expressing splenocytes were i.v. transferred at 24 h after DT injection, and the positioning of donor splenocytes in the omentum was determined by fluorescence imaging. At 6 h after transfer, donor splenocytes in WT mice abundantly accumulated in the region of milky spots with only a few GFP^+^ cells scattered in the surrounding regions (Fig. 4 F). Meanwhile, milky spots of *Postn*-DTR mice had significantly fewer numbers of GFP^+^ splenocytes. Consistently, the numbers of GFP^+^ cells in omentum, but not in inguinal lymph node, significantly reduced in *Postn*-DTR mice (Fig. 4 G). Collectively, these data indicate that ablation of *Aldh1a2*^+^ FRCs led to the selective impairment of lymphocyte recruitment to the milky spots. Interestingly, majority of F4/80^+^ macrophages were found outside of the lymphoid aggregates that were marked with B220 (Fig. S4 G), suggesting that the conventional lymphocytes are continuously recruited to the milky spots whereas other leukocytes such as macrophages reside primarily outside of the milky spots.

### *Aldh1a2*^+^ FRCs are required for the induction of CXCL12

**Figure 5.**
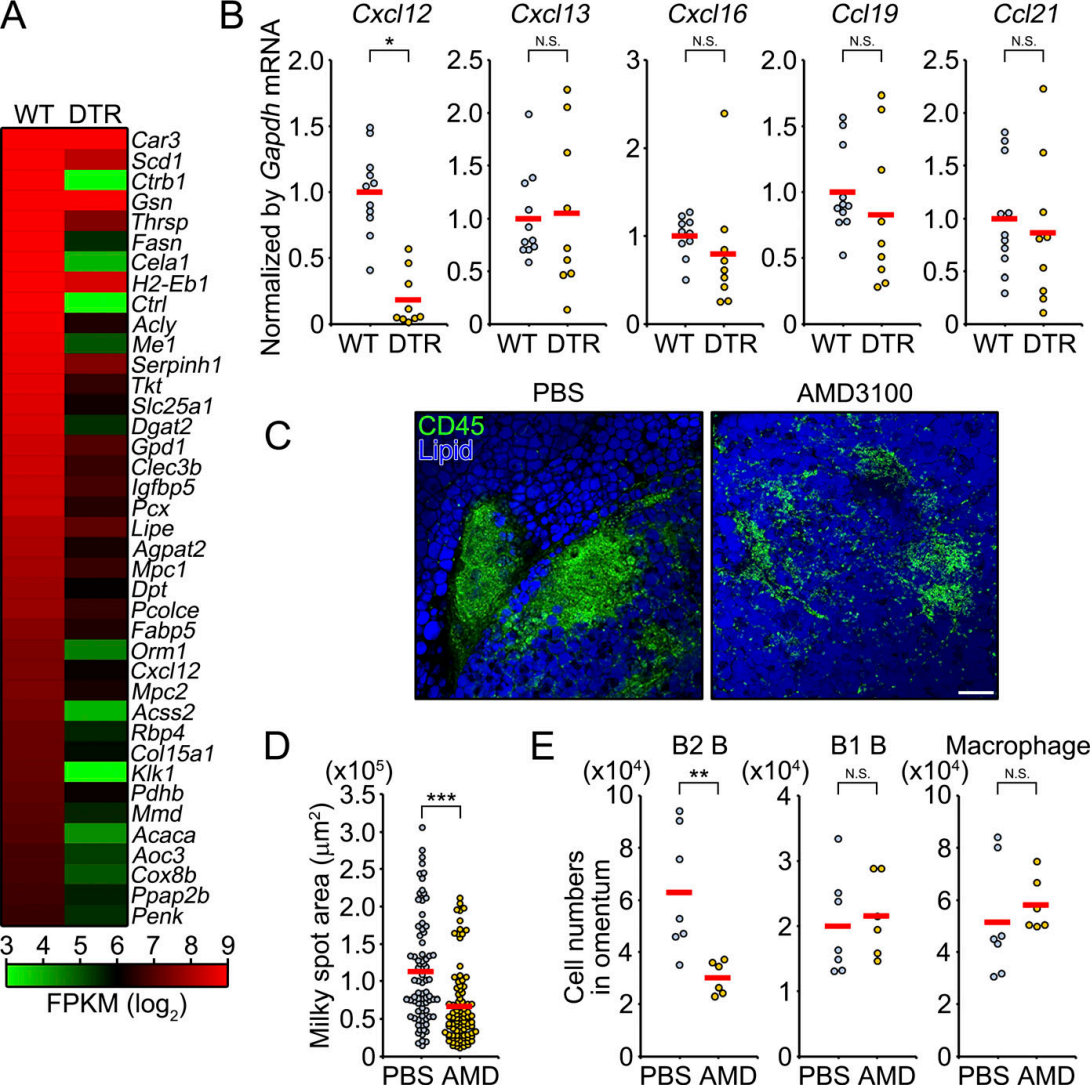
***Aldh1a2***^**+**^ **FRCs are required for *Cxcl12* expression. (A)** Heat map of mRNA, as measured by RNA-seq, expressed at least two times over in omenta of WT mice relative to their expression in that of *Postn*-DTR mice at 5 d after DT injection. Expression levels were shown as fragments per kilobase of exon per million reads mapped (FPKM, log_2_). **(B)** The expression of indicated chemokines in WT and *Postn*-DTR omenta was analyzed by quantitative PCR and expressed as relative values normalized by *Gapdh* mRNA. Each point represents one mouse, and the mean values were shown by red horizontal lines (*n* = 9–11). **(C)** Representative images of omenta from WT mice that were injected with PBS or AMD3100. **(D)** The area of milky spots of C was determined from cumulative vertical images. Each plot represents one CD45^+^ lymphoid aggregate. Red horizontal lines indicate the mean. **(E)** PBS or AMD3100 was i.p. injected every day for 5 d, and numbers of B2 B, B1 B, and macrophages in the omentum were determined. Each plot represents one mouse, and red horizontal lines indicate the mean. Pooled data from at least two independent experiments (B, D, and E) or the representative image of at least two independent experiments with similar results (C) are shown. P value <0.05 was considered significant (*, P < 0.05; **, P < 0.01; ***, P < 0.001). N.S., not significant. Student’s *t* test was used. Scale bar: 100 μm (C).

The above data suggested that *Aldh1a2*^+^ FRCs directly or indirectly recruit circulating lymphocytes to the milky spots. To delineate the molecular mechanism underlying lymphocyte recruitment, we performed RNA-seq analysis of omenta at 5 d after DT injection and identified 39 genes that were downregulated at least 0.5-fold in *Postn*-DTR mice compared with WT mice (Fig. 5 A). Among these genes, chemokine *Cxcl12*, which recruits a diverse range of CXCR4-expressing leukocytes (Campbell et al., 2003), was significantly downregulated in omenta of *Postn*-DTR mice, whereas other homeostatic chemokines such as *Cxcl13*, *Cxcl16*, *Ccl19*, and *Ccl21* were comparably expressed between omenta of WT and *Postn*-DTR mice (Fig. 5 B). Given that CXCL12 is involved in the formation of ectopic lymphoid tissues (Fleige et al., 2014) and its receptor CXCR4 was broadly expressed in omental lymphocytes including T cells and B2 B cells (Fig. S4 C), we investigated the functional role of *Cxcl12* in milky spot formation. To this end, WT C57BL/6 mice were i.p. injected with AMD3100, a specific antagonist of CXCR4, and the size of the milky spots was determined by immunofluorescence staining for CD45. We observed that the administration of AMD3100 significantly reduced the size of milky spots (Fig. 5, C and D), consistent with the previous observation (Abe et al., 2009). Additionally, the administration of AMD3100 reduced the numbers of B2 B cells, but not B1 B cells and macrophages, in omentum (Fig. 5 E), similar to those seen in *Postn*-DTR mice. Collectively, these results indicate that *Aldh1a2*^+^ FRCs are required for the induction of CXCL12 which is, at least in part, the mechanism regulating milky spot formation.

### Retinoic acid–dependent induction of CXCL12 on HEVs

**Figure 6.**
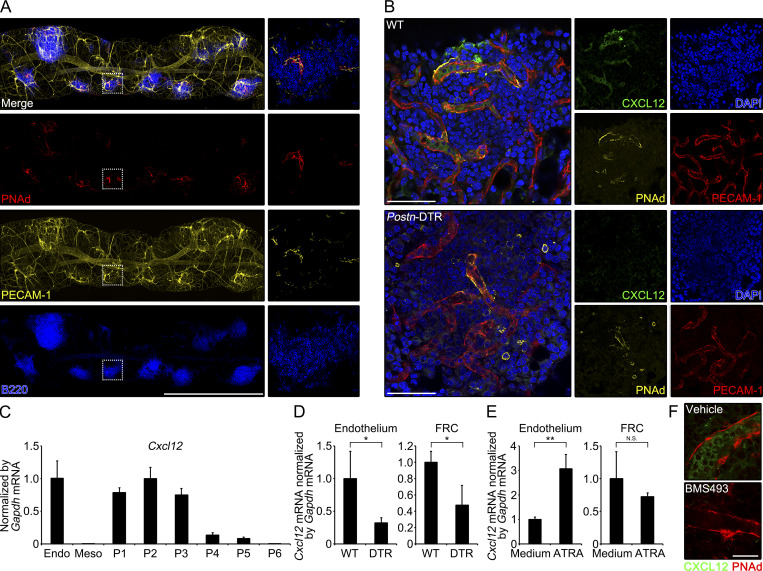
**Involvement of *Aldh1a2***^**+**^ **FRCs in CXCL12 induction in HEVs. (A)** Immunofluorescence staining of whole-mount omentum of WT mouse demonstrating the expression of PNAd, PECAM-1, and B220. Right: Enlarged images of boxed regions. **(B)** Milky spot of the whole-mount omunta from WT and *Postn*-DTR mice was analyzed for CXCL12, PNAd, PECAM-1, and DAPI. **(C)** The expression of *Cxcl12* mRNA in sorted endothelial cells (Endo), mesothelial cells (Meso), and P1–P6 was determined by quantitative PCR and expressed as relative values normalized by *Gapdh* mRNA (*n* = 3). **(D)** The expression of *Cxcl12* mRNA in sorted endothelial cells and FRCs in WT and *Postn*-DTR mice was determined by quantitative PCR and expressed as relative values normalized by *Gapdh* mRNA (*n* = 3). **(E)** Sorted endothelial cells and FRCs were stimulated with 1 μM ATRA for 24 h and the expression of *Cxcl12* mRNA was quantified (*n* = 3). **(F)** Representative immunofluorescence images of omenta from WT mice that were injected with vehicle or BMS493. Data are representative of at least two independent experiments (A, B, and D–F). P value <0.05 was considered significant (*, P < 0.05; **, P < 0.01). N.S., not significant. Student’s *t* test was used. Scale bar: 1 mm (A), 50 μm (B), 20 μm (F).

**Figure S5.**
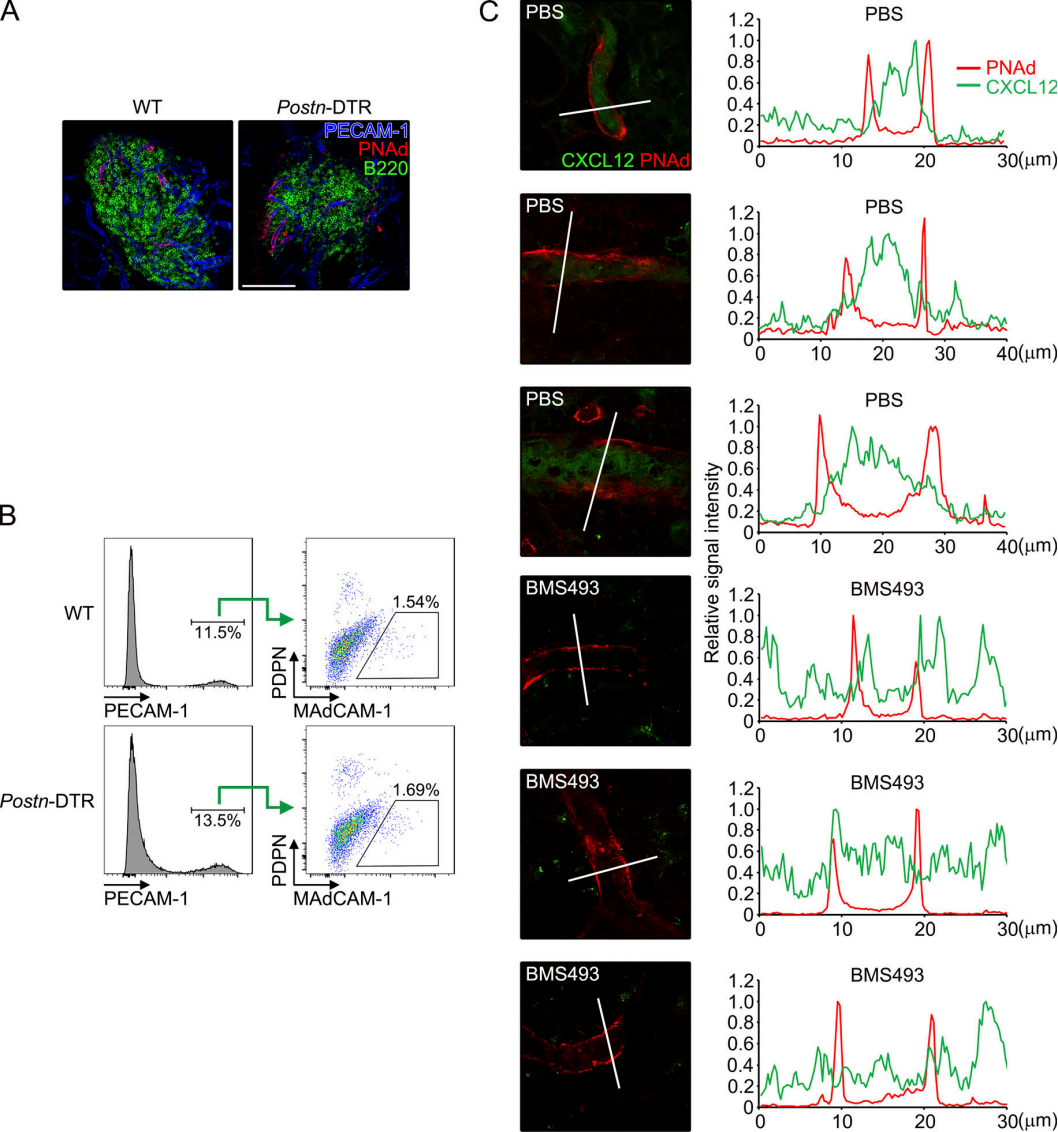
**Characterization of HEVs in *Postn*-DTR mice. (A and B)** WT and *Postn*-DTR mice were i.p. injected with 0.1 μg DT. Whole-mount omenta from indicated mice were stained as indicated color-coded lettering (A). Omenta were analyzed by flow cytometry (B). **(C)** Left: Three representative images for each treatment (PBS or BMS493) in Fig. 6 F are shown. Right: The signal intensity within the line (white) on the left was quantified every 0.026 μm for PNAd (red) and CXCL12 (green), and that relative to the maximum intensity was depicted. Scale bar: 100 μm (A).

We next addressed CXCL12-producing cells in omentum. It has been shown that HEVs in lymph nodes display CXCL12, which promotes extravasation of blood-borne leukocytes into the lymph node parenchyma (Okada et al., 2002; Pablos et al., 2003; Santiago et al., 2006), and these observations prompted us to determine the localization of CXCL12 on omental HEVs. Consistent with the previous studies (Berberich et al., 2008; Rangel-Moreno et al., 2009), PECAM-1^+^ vessels expressing HEV marker, peripheral node addressin (PNAd), were exclusively present inside milky spots (Fig. 6 A). Additionally, we observed predominant localization of CXCL12 in the luminal side of HEVs in WT milky spots (Fig. 6 B). In contrast, CXCL12 was absent on HEVs in omenta of *Postn*-DTR mice (Fig. 6 B), despite PECAM-1^+^ cells expressing PNAd and another HEV marker, mucosal addressin cell adhesion molecule 1 (MAdCAM-1), were equally present in omenta of WT and *Postn*-DTR mice (Fig. S5, A and B). CXCL12 on HEVs can be produced by endothelial cells themselves or can be produced by surrounding stromal cells and, subsequently, transported to HEVs by transcytosis (Rustenhoven et al., 2021; Weiss et al., 2013). The expression of *Cxcl12* mRNA in omentum was found in PECAM-1^+^ endothelial cells, *Aldh1a2*^+^ FRCs (P1), and conventional FRCs (P2 and P3; Fig. 6 C). Additionally, the expression of Cxcl12 mRNA in both endothelial cells and FRCs was downregulated in *Postn*-DTR mice (Fig. 6 D). Thus, the decline of *Cxcl12* expression in whole omental tissues of *Postn*-DTR mice could be reflected by the reduction in gene expression in endothelium and conventional FRCs and the ablation of *Aldh1a2*^+^ FRCs.

Multiple studies have shown that retinoic acid induces the expression of *Cxcl12* (Liu et al., 2011; Hu et al., 2017; Kim et al., 2007), suggesting the possibility that *Aldh1a2*^+^ FRC-derived retinoic acid induces the expression of *Cxcl12* gene in endothelial cells and/or FRCs. To test this possibility, we sorted PECAM-1^+^ endothelial cells and PDPN^+^CD140a^+^ FRCs from omenta of WT C57BL/6 mice and stimulated with all trans retinoic acid (ATRA), the most abundant form of retinoic acid in vivo, for 24 h. We observed ATRA stimulation increased the expression of *Cxcl12* in endothelial cells about 3.1-fold, whereas it had only marginal effect on the expression in FRCs (Fig. 6 E). To further explore the role of retinoic acid in the expression of omental *Cxcl12* gene in vivo, WT C57BL/6 mice were i.p. injected with pan-retinoic acid receptor inverse agonist BMS493. Interestingly, BMS493 treatment was able to diminish the CXCL12 display on HEVs in milky spots (Fig. 6 F and Fig. S5 C). Altogether, these results suggest that homeostatic display of CXCL12 on HEVs in the milky spots is primarily regulated by retinoic acid–dependent induction of *Cxcl12* gene in endothelium.

### *Aldh1a2*^+^ fibroblastic stromal cells maintain peritoneal leukocyte population

**Figure 7.**
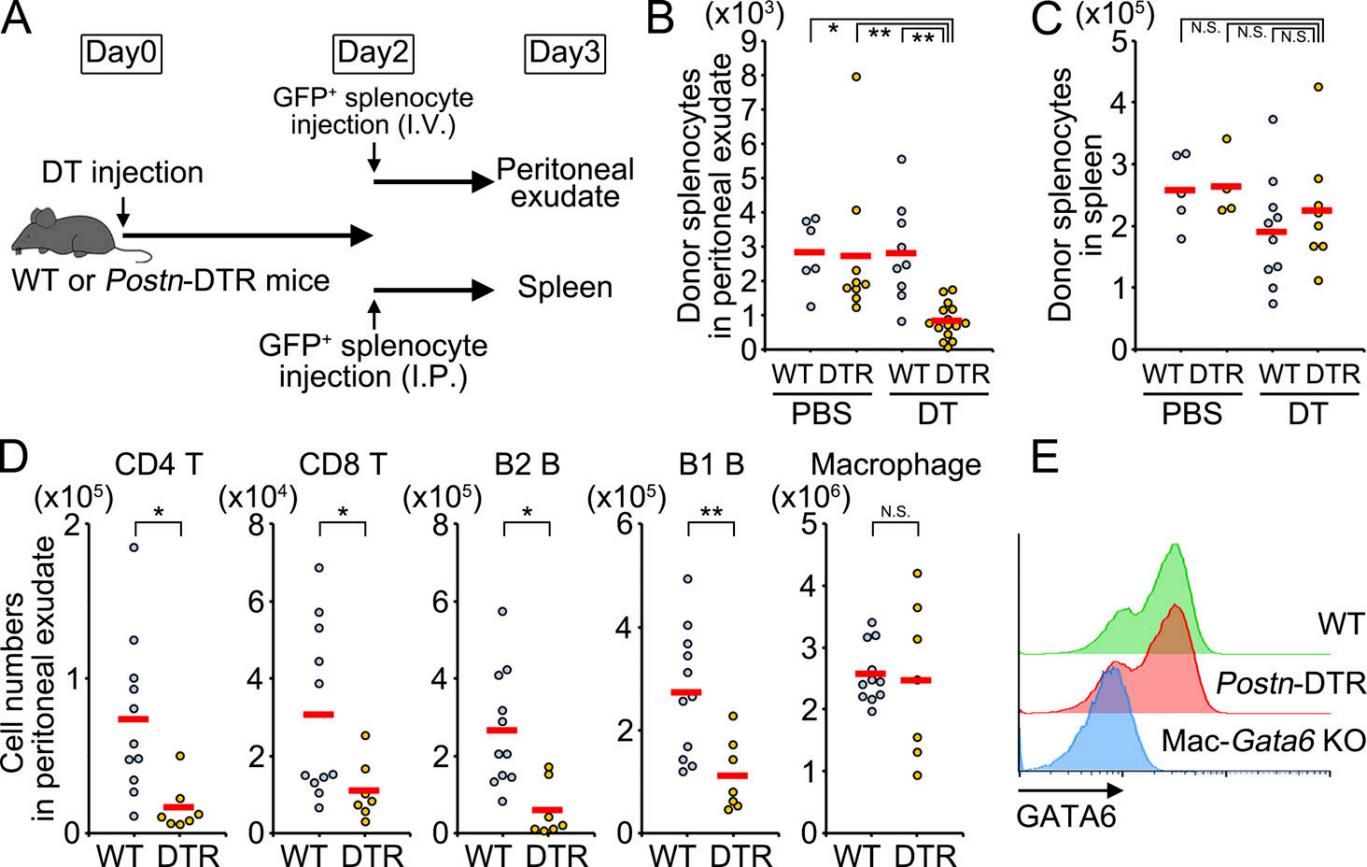
**Requirement of *Aldh1a2***^**+**^ **FRCs for peritoneal lymphocyte population. (A–C)** Schematic of splenocyte transfer. GFP^+^ splenocytes were i.v. or i.p. transferred into WT or *Postn*-DTR mice 2 d after PBS or DT injection and the numbers of GFP^+^ cells in the peritoneal exudate (B) and spleen (C) were shown. Each plot represents one mouse, and red horizontal lines indicate the mean. **(D)** Mean numbers of CD4 T, CD8 T, B2 B, B1 B cells, and macrophages in the peritoneal cavity from WT and *Postn*-DTR mice at 6 d after DT injection. **(E)** Large peritoneal macrophages in peritoneal exudate cells in D and Mac-*Gata6* KO mice were analyzed for GATA6 protein by flow cytometry. Pooled data from at least two independent experiments (B–D) or the representative flow cytometry of at least two independent experiments with similar results (E) are shown. P value <0.05 was considered significant (*, P < 0.05; **, P < 0.01). N.S., not significant. One-way ANOVA was used for B and C, and Student’s *t* test was used for D.

Given that the milky spots of omentum are proposed as the source of peritoneal cells (Ansel et al., 2002; Barington et al., 2022), we next explored the role of *Aldh1a2*^+^ FRCs in peritoneal lymphocyte composition. To this end, GFP-expressing splenocytes were adoptively transferred i.v. or i.p. at 2 d after DT injection, and the number of donor cells in peritoneal cavity or spleen was determined by flow cytometry at 24 h after transfer, respectively (Fig. 7 A). Although the depletion of *Aldh1a2*^+^ FRCs did not affect the lymphocyte homing from peritoneal cavity to spleen, that from circulation to peritoneal cavity was significantly diminished (Fig. 7, B and C). Consistently, flow cytometry of peritoneal lavage cells at 6 d after DT injection revealed that *Postn*-DTR mice had 4.4-fold fewer CD4 T cells, 2.8-fold fewer CD8 T cells, and 4.5-fold fewer B2 cells compared with that of WT controls (Fig. 7 D). These results indicate that *Aldh1a2*^+^ FRCs are required for the recruitment of T cells and B2 cells to peritoneal cavity.

The reduction in T cell and B2 cell numbers in *Postn*-DTR mice seemed to be primarily caused by poor lymphocyte recruitment to the milky spots (Fig. 4, E and G). In addition to these conventional lymphocytes, *Postn*-DTR mice had 2.4-fold fewer B1 B cells in the peritoneal cavity (Fig. 7 D), whereas the number of omental B1 B cells was not affected in *Postn*-DTR mice (Fig. 4 E). This suggests the possibility that DT had some impact on the lymphocyte egress to the peritoneal cavity from omentum or other sources (Berberich et al., 2008; Jia et al., 2020). In contrast, the number of peritoneal resident cells such as macrophages was not affected in *Postn*-DTR mice (Fig. 7 D). Consistently, the expression of GATA6, which regulates tissue-specific transcription program and localization of peritoneal macrophages (Okabe and Medzhitov, 2014), was not affected in CD11b^+^ F4/80^+^ MHC-II^−^^ or low^ large peritoneal macrophages of *Postn*-DTR mice (Fig. 7 E). These results indicate that *Aldh1a2*^+^ FRCs were not essential for macrophage polarization and localization in peritoneal cavity.

## Discussion

In the present study, we identified a subset of FRCs that was uniquely present in omental milky spots. They were characterized by the enriched expression of gene-encoding retinoic acid converting enzyme, *Aldh1a2*, as well as endothelial cell–associated genes, including *Tie2*. Ablation of *Aldh1a2*^+^ FRCs was not immediately accompanied by the collapse of the lymphoid aggregates, and population of B1 B cells and macrophages was maintained, which suggested that lymphoid infrastructure was maintained despite a lack of *Aldh1a2*^+^ FRCs. In contrast, the ablation of *Aldh1a2*^+^ FRCs was associated with the impaired recruitment of circulating lymphocytes to the milky spots, and our results indicated *Aldh1a2*^+^ FRCs are required for display of CXCL12 on HEVs. Thus, *Aldh1a2*^+^ FRCs are an integral component of milky spot formation, which is mediated by the recruitment of circulating lymphocytes in a manner dependent on CXCL12.

**Figure 8.**
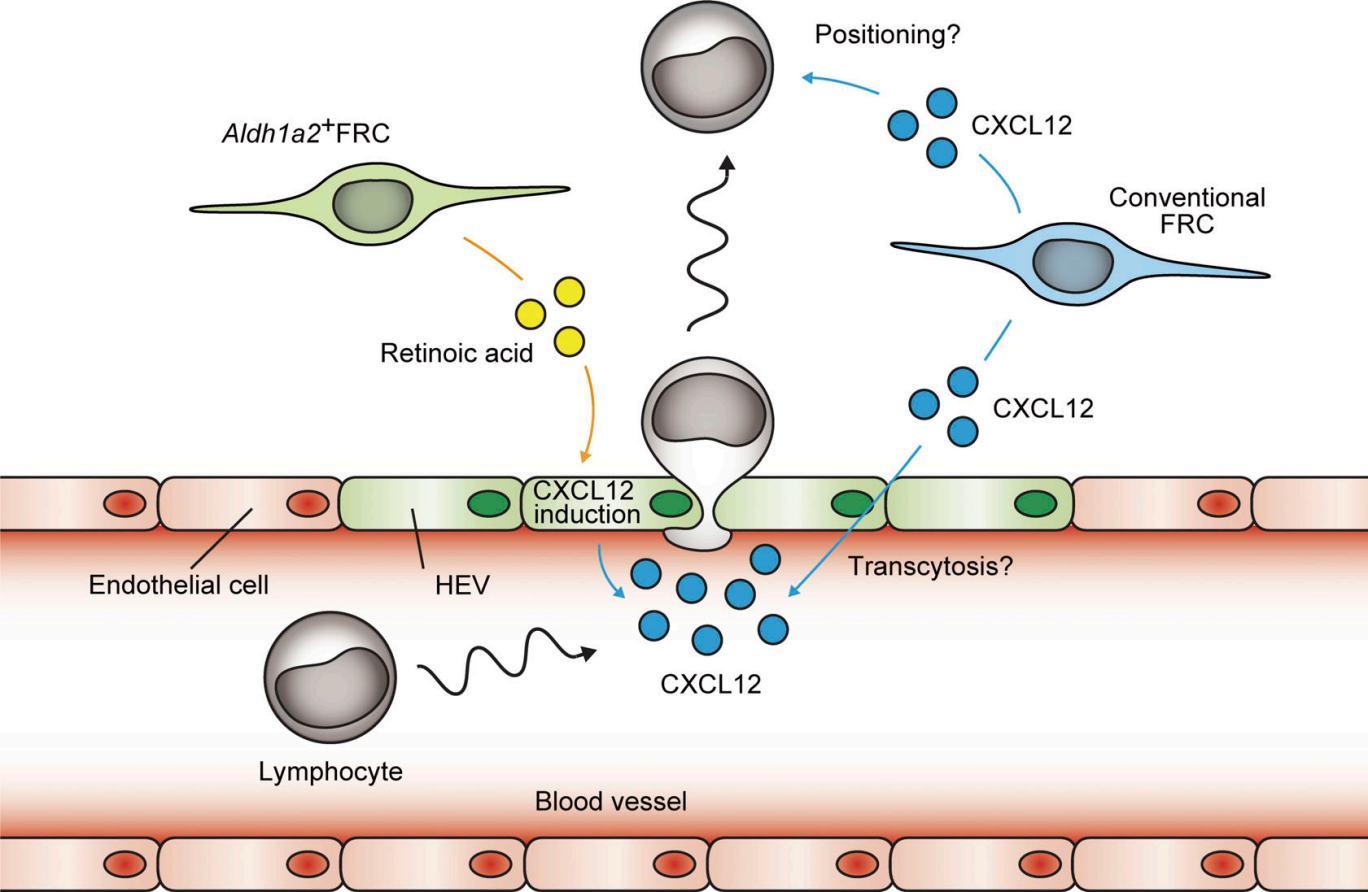
**Proposed model for recruitment of circulating lymphocytes into the milky spots.** CXCL12 in the milky spots is produced from two sources. *Aldh1a2*^+^ FRC-derived retinoic acid induces the expression of CXCL12 in endothelium, which regulates the constitutive recruitment of circulating lymphocytes to the milky spots. Additionally, conventional FRCs are also the source of CXCL12.

Although retinoic acid is abundantly present in omentum under homeostatic conditions, the role of retinoic acid in the development and maturation of milky spots remained underexplored. In lymph nodes and Peyer’s patches, retinoic acid initiates lymphoid organ development through the induction of homeostatic chemokine CXCL13 in stromal organizer cells (van de Pavert et al., 2009). However, the ablation of *Aldh1a2*^+^ FRCs in omentum did not alter *Cxcl13* expression (Fig. 5 B). By contrast, our results indicated *Aldh1a2*^+^ FRC-derived retinoic acid induced CXCL12 in endothelial cells, which is essential for constitutive recruitment of circulating lymphocytes into the milky spots. On the opposite side, *Cxcl12* mRNA was also expressed in omental FRCs in a manner independent of retinoic acid. CXCL12 is a key regulator for the development of ectopic lymphoid follicles and, notably, CXCL12 in FRCs is induced by inflammatory signals such as IL-17 during the development of bronchus-associated lymphoid tissue (Fleige et al., 2014; Pandey et al., 2017; Meza-Perez and Randall, 2017). Collectively, this evidence suggest that retinoic acid–dependent CXCL12 induction in endothelium preserves the milky spot formation under homeostatic conditions, whereas FRC-derived CXCL12 might be rather prone to the expansion of lymphoid structure during the inflammatory settings (Fig. 8).

Retinoic acid is essential for the maintenance of peritoneal macrophages through the induction of transcription factor GATA6 (Okabe and Medzhitov, 2014). However, we did not observe a significant change in the peritoneal macrophage number as well as GATA6 expression in *Postn*-DTR mice (Fig. 7, D and E). A previous study has shown that Wilms’ tumor 1 (WT1)–expressing omental stromal cells, including fibroblasts and mesothelial cells, are the source of retinoic acid, and depletion of WT1^+^ cells reduced the frequency of GATA6^+^ macrophages in the peritoneal cavity (Buechler et al., 2019). We found that the expression of *Wt1* mRNA was rather lower in *Aldh1a2*^+^ FRCs among the stromal populations (Fig. S2 B), suggesting WT1^+^ cells and *Aldh1a2*^+^ FRCs are distinct populations. Given that mesothelium is in particular the first contact with peritoneal macrophages that drift with peritoneal fluid flow, mesothelial cell–derived retinoic acid may be prone to the maintenance of peritoneal macrophage population. Collectively, distinct sources of retinoic acid, *Aldh1a2*^+^ FRCs and WT1^+^ mesothelial cells, may endow the distinct regulating mechanisms in omental biology.

*Postn* gene was selectively expressed in *Aldh1a2*^+^ FRCs among omental stromal populations (Fig. 2 B). Indeed, DT administration in *Postn*-DTR mice selectively impaired the lymphocyte recruitment to the milky spots, but not to the other lymphoid organs (Fig. 4 G and Fig. S4 F). However, *Postn* expression was reported in cells of mesenchymal lineage present in other tissues, such as stomach, colon, and bone (Kanisicak et al., 2016; Morra and Moch, 2011). We observed that some *Postn*-DTR mice, after DT injection, exhibited cecal enlargement that might be caused by tissue damage in stomach and/or colon. Therefore, systemic effects of *Postn*-expressing cell ablation on the milky spot formation cannot be ruled out. Additionally, this made it difficult to study long term effect of *Aldh1a2*^+^ FRC ablation. Although this study illuminated the role of *Aldh1a2*^+^ FRCs in omentum and peritoneal cavity, these immune compartments also contribute to building systemic immunity including the production of serum natural antibodies and intestinal IgA (Ansel et al., 2002; Kunisawa et al., 2007). Further study is needed regarding the roles of *Aldh1a2*^+^ FRCs in these immune responses.

## Materials and methods

### Animals

All mice were bred in specific pathogen–free conditions, and experiments were approved by the Institutional Animal Care and Use Committee of Osaka University. Littermate controls were used for all experiments when feasible. C57BL/6J mice were purchased from CLEA Japan. CAG-EGFP mice (RBRC 00267) were obtained from Riken BioResource Research Center (Okabe et al., 1997), Mac-*Gata6* KO was described previously (Okabe and Medzhitov, 2014), and these mice were backcrossed to B6. *Postn*-DTR mice were generated by CRISPR/Cas-mediated gene targeting in C57BL/6 zygotes. In brief, CRISPR RNA: 5′-ACG​GAG​CTC​AGG​GCT​GAA​GA-3′ was used as targeting guide (Integrated DNA technologies) and mutated DTR sequence was inserted by microinjection (Saito et al., 2001; Furukawa et al., 2006).

### Reagents and antibodies

Rabbit monoclonal antibodies against ALDH1A2 (E6O6Q; Cell Signaling Technology), Desmin (Y66; Abcam), and CXCL12 (79018; R&D) were used. Antibodies against B220 (RA3-6B2), CD3 (17A2), CD4 (GK1.5), CD8a (53-6.7), CD11b (M1/70), CD16/32 (93), CD19 (6D5), CD23 (B3B4), CD45 (30-F11), CD105 (MJ7/18), F4/80 (BM8), IgM (RMM-1), MAdCAM-1 (MECA-367), PECAM-1 (390), PNAd (MECA-79), PDPN (8.1.1), TIE2 (TEK4), VCAM-1 (429), and Rat IgG2a,κ isotype control (RTK2758) were obtained from BioLegend. Antibodies against CD140a (APA5) and Msln (295D) were obtained from BD Biosciences and MDL, respectively. ATRA was obtained from Sigma-Aldrich.

For DT-mediated cell ablation, mice were i.p. injected with 100 ng of DT (Sigma-Aldrich). For CXCR4 inhibition, 200 μg of AMD3100 (Sigma-Aldrich) was i.p. injected every day for five times and omenta were harvested 24 h after the last injection. For inhibition of retinoic acid signaling, 500 nmol of pan retinoic acid receptor inverse agonist BMS493 (Tocris) was i.p. injected every day for three times and omenta were harvested 24 h after the last injection.

### Flow cytometry

Tissues were separated, minced, and incubated in PBS containing 0.2 U/ml of Liberase TM (Roche) and 20 μg/ml DNase I (Roche) in the presence of calcium and magnesium. After digestion, the suspension was further mechanically disrupted by pipetting and filtered through 70-μm cell strainer. Peritoneal exudate cells were harvested by injecting 8 ml of PBS containing 10% fetal bovine serum and 2 mM EDTA into peritoneal cavity. Single-cell suspensions were preincubated with antibody against CD16/32 to block FcγRII/III receptors and stained on ice for 10 min with antibodies conjugated with fluorochrome. Flow cytometry was performed on an LSRFortessa (BD Biosciences), and aldehyde dehydrogenase activity was determined by ALDEFLUOR (StemCell Technologies).

### RNA extraction and real-time PCR

Total RNA was reverse-transcribed using PrimeScript RT Master Mix (Clonetech) containing random hexamer and oligo (dT) primer. Alternatively, SMART MMLV Reverse Transcriptase (Clonetech) with oligo (dT) primer was used. Aliquots of the products were amplified using BioRad CFX96. The primers used for the mouse genes were as follows: *Gapdh*, 5′-GTT​GTC​TCC​TGC​GAC​TTC​AAC-3′ and 5′-CCA​GGG​TTT​CTT​ACT​CCT​TGG-3′; *Rpl13a*, 5′-GGC​TGA​AGC​CTA​CCA​GAA​AGT-3′ and 5′-TCT​TTT​CTG​CCT​GTT​TCC​GTA-3′; *Aldh1a2*, 5′-CAC​AGG​AGA​GCA​AGT​GTG​TGA-3′ and 5′-TAG​TTG​CAA​GAG​TTG​CCC​TGT-3′; *Aldh1a1*, 5′-GCA​CTC​AAT​GGT​GGG​AAA​GT-3′ and 5′-TTT​GGC​CAC​ACA​CTC​CAA​TA-3′; *Aldh1a3*, 5′-AAA​CCC​ACG​GTC​TTC​TCA​GAT-3′ and 5′-CTT​TGT​CCA​GGT​TTT​TGG​TGA-3′; *Phex*, 5′-GGG​TTT​ATC​CTT​GGC​TGA​GAC-3′ and 5′-AGG​TGA​ATG​CCT​CAA​GAT​GTG-3′; *Postn*, 5′- AAC​CAA​GGA​CCT​GAA​ACA​CG-3′ and 5′- GTG​TCA​GGA​CAC​GGT​CAA​TG-3′; *Ptx3*, 5′-CCT​GCT​TTG​TGC​TCT​CTG​GT-3′ and 5′-TCT​CCA​GCA​TGA​TGA​ACA​GC-3′; *Cd143*, 5′-CAG​TGT​CTA​CCC​CCA​AGC​AT-3′ and 5′-TTC​CAT​CAA​AGA​CCC​TCC​AG-3′; *Adm*, 5′-GTC​GTG​GGA​AGA​GGG​AAC​TAC-3′ and 5′-GGT​AGC​GTT​TGA​CAC​GAA​TGT-3′; *Tie2*, 5′-CAG​GCC​TGG​AAA​TAC​ATT​GAA-3′ and 5′-GGC​AGG​AGA​CTG​AGA​CCT​CTT-3′; *Msln*, 5′-AGT​CAG​GGA​GGT​TCT​GAG​GAG-3′ and 5′-AGG​GGT​CTC​TGG​AGA​TGT​GTT-3′; *Lrrn4*, 5′-TGA​GTT​CCT​TTG​GTC​CTT​GG-3′ and 5′-TAA​AGC​AGG​CTC​ACA​CAT​GG-3′; *Upk3b*, 5′-AGA​ATC​CCA​ACT​CCA​TTG​ACA​C-3′ and 5′-ATG​TAA​CGT​TTT​CCC​ATG​AAG​G-3′; *Ccl19*, 5′-CAC​CAC​ACT​AAG​GGG​CTA​TCA-3′ and 5′-TCT​TCT​GGT​CCT​TGG​TTT​CCT-3′; *Col4a1*, 5′-GAG​AGA​CAG​GAC​CCT​TTG​GAC-3′ and 5′-GGA​CAC​AGT​GGG​TCA​TCT​GTT-3′; *Col14a1*, 5′-CTG​CCC​ACA​CAG​CTA​GTG​AA-3′ and 5′-GCC​AGA​CCT​TCT​GTG​AGA​GG-3′; *Cxcl12*, 5′-GCT​CTG​CAT​CAG​TGA​CGG​TA-3′ and 5′-TAA​TTT​CGG​GTC​AAT​GCA​CA-3′; *Cxcl13*, 5′-TCT​GGA​AGC​CCA​TTA​CAC​AA-3′ and 5′-TTT​GTA​ACC​ATT​TGG​CAC​GA-3′; *Cxcl16*, 5′-TCC​GTG​AAC​TAG​TGG​ACT​GCT-3′ and 5′-GGA​AGA​GTG​GAG​TGC​TGA​GTG-3′; *Ccl21*, 5′-ATG​TGC​AAA​CCC​TGA​GGA​AG-3′ and 5′-TCC​TCT​TGA​GGG​CTG​TGT​CT-3′; *Dpt*, 5′-CCA​CTA​TGG​GGA​AGA​CAT​GG-3′ and 5′-CCT​TCA​CCC​GGA​CTT​CTG​TA-3′; *Wt1*, 5′- ACA​CCA​AAG​GAG​ACA​CAC​AGG-3′ and 5′- GCG​CAA​ACT​TTT​TCT​GAC​AAC-3′.

### RNA-seq

Omental cells were sorted by FACS Aria III (BD Biosciences) as indicated in the specific experiments. RNA was purified using RNeasy Micro Kit (QIAGEN) and RNAprotect Cell Reagent (QIAGEN). Sequencing libraries were constructed using NEBNext Ultra RNA Library Prep Kit for Illumina (NEB), and single-end sequencing was performed with Illumina NextSeq 500 with 76 bp reads. Gene expression heat map was created by using Cluster 3.0 and Java TreeView software. The accession numbers for the RNA-seq dataset reported in this paper are GEO GSE224315 and GSE224023.

### scRNA-seq

Single-cell suspension isolated from the omenta from three mice per group (WT and *Postn*-DTR, 5 d after DT injection) were run on 10X chromium (10X Genomics) and then through library preparation following the recommended protocol for the Chromium Single Cell 3′ Reagent Kit (v2). Fragment size of the libraries was confirmed with an Agilent 2100 Bioanalyzer. Libraries were sequenced on Illumina NovaSeq 6000 as a single-end mode. The raw reads were processed by Cell Ranger 3.1.0 (10X Genomics). Gene expression–based clustering was performed using the Seurat R package and filtering was applied to remove low-quality cells by excluding cells expressing <200 or >7,500 unique genes, as well as cells with >25% mitochondrial gene expression. The Seurat SCTransform function was used for normalization, and data were integrated without performing batch-effect correction as all samples were processed simultaneously. The accession number for the scRNA-seq dataset reported in this paper is GEO GSE224483.

### Splenocyte transfer

Single-cell suspensions of splenocytes from CAG-EGFP mice were prepared by homogenization of the spleen by using the frosted ends of the glass slides, followed by the filtration through 70-μm cell strainer. 5 × 10^7^ cells of splenocytes were injected i.v. or i.p., and peritoneal exudates or spleens were harvested after 24 h injection, respectively.

### Histology, immunofluorescence, and image analysis

For immunofluorescence of whole-mount tissues, omenta were dissected and fixed with 4% paraformaldehyde and blocked with PBS containing 5% bovine serum albumin in the presence of 2 μg/ml of antibody against CD16/32 (BioLegend). Specimens were stained with Can Get Signal immunostain Immunoreaction Enhancer Solution A (Toyobo) containing antibodies and mounted with Prolong Glass (Thermo Fisher Scientific). Samples were analyzed with a confocal laser scanning microscope (Leica STELLARIS 5 or Olympus FV-3000). Images were created using Leica Application Suite X or Olympus FV31S-SW.

The milky spot size was semiautomatically determined by BZ-X800 analyzer (Keyence). Recruitment of GFP^+^ splenocytes into the milky spots was statistically analyzed by using BZ-X800 analyzer (Keyence). The number of GFP signals in full-focused images from the multiple sectioning images (0.4 μm pitch) was automatically counted by BZ-X800 analyzer.

### Statistical analysis

All experiments were performed at least twice. Results were statistically analyzed by an ANOVA test or Student’s *t* test using GraphPad Prism 9. No statistical methods were used to predetermine sample size. Mean ± SD is shown for bar graphs, and P values of <0.05 were considered statistically significant. Sample sizes are detailed in the figure legends.

### Online supplemental material

Fig. S1 shows the expression of *Aldh1a1* gene among tissues, cellular characterization of ALDH-producing cells in omentum, and localization of ALDH1A2^+^ cells in the omentum. Fig. S2 shows gene expression analysis of *Aldh1a2*^+^ FRCs. Fig. S3 shows targeting construct of *Postn*-DTR allele and population analysis of omental stromal fractions. Fig. S4 includes Desmin^+^ FRC network, scRNA-seq analysis, and lymphoid organization of WT and *Postn*-DTR mice. Fig. S5 shows HEV populations in WT and Postn-DTR omenta and quantification of CXCL12 signal in the lumen of HEVs.
